# Laboratory measurements of resistivity in warm dense plasmas relevant to the microphysics of brown dwarfs

**DOI:** 10.1038/ncomms9742

**Published:** 2015-11-06

**Authors:** N. Booth, A. P. L. Robinson, P. Hakel, R. J. Clarke, R. J. Dance, D. Doria, L. A. Gizzi, G. Gregori, P. Koester, L. Labate, T. Levato, B. Li, M. Makita, R. C. Mancini, J. Pasley, P. P. Rajeev, D. Riley, E. Wagenaars, J. N. Waugh, N. C. Woolsey

**Affiliations:** 1 Central Laser Facility, STFC Rutherford Appleton Laboratory, Didcot OX11 0QX, UK; 2 Department of Physics, College of Science, University of Nevada, Reno, Nevada 89557-0208, USA; 3 Department of Physics, York Plasma Institute, University of York, Heslington York YO10 5DD, UK; 4 School of Mathematics and Physics, Queen's University Belfast, Belfast BT1 4NN, UK; 5 Intense Laser Irradiation Laboratory, Istituto Nazionale di Ottica, Area della Ricerca del CNR, 56124 Pisa, Italy; 6 Department of Physics, University of Oxford, Oxford OX4 3PU, UK

## Abstract

Since the observation of the first brown dwarf in 1995, numerous studies have led to a better understanding of the structures of these objects. Here we present a method for studying material resistivity in warm dense plasmas in the laboratory, which we relate to the microphysics of brown dwarfs through viscosity and electron collisions. Here we use X-ray polarimetry to determine the resistivity of a sulphur-doped plastic target heated to Brown Dwarf conditions by an ultra-intense laser. The resistivity is determined by matching the plasma physics model to the atomic physics calculations of the measured large, positive, polarization. The inferred resistivity is larger than predicted using standard resistivity models, suggesting that these commonly used models will not adequately describe the resistivity of warm dense plasma related to the viscosity of brown dwarfs.

## Abstract (web summary)

Brown dwarfs are small stars that are believed to be made of a warm dense plasma that cannot support hydrogen fusion as larger stars do. Here, the authors present a method for studying the properties, such as resistivity, of warm dense plasmas in the laboratory.

## Graphical abstract

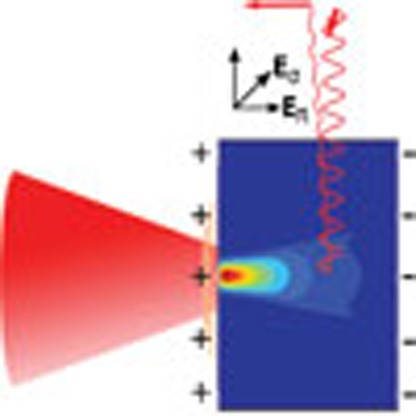

Intense laser interactions with matter provide us with the ability to probe the conditions present at the core of dense stars in the laboratory^1^. Brown dwarfs are an ideal candidate for study with laboratory plasmas as their cores are at temperatures and mass densities of ∼2–3 × 10^6^ K (∼200 eV) and 10^2^–10^3^ g cm^−3^, respectively^2^, which are comparable to conditions in laser-produced plasmas. The viscosity and equation of state of these systems are uncertain and requires detailed modelling^3^. The heat transport and electron propagation models at high density and pressure are complex^4^,^5^,^6^,^7^ and need benchmarking. Laser-produced plasmas are a method of creating matter at conditions that approach those believed to occur in brown dwarfs^8^,^9^ enabling laboratory studies of the plasma in these systems^10^.

Plasma polarization spectroscopy is an *in-situ* method for probing the resistivity inside warm dense plasma environments^11^,^12^,^13^. The technique is sensitive to the complex electron microphysics of collisions in a solid, allowing us to observe the non-equilibrium states in the electron distributions. Other measurements have provided an indirect method of examining the sensitivity of electron transport to low-temperature resistivity in thick, cold, targets (for example, refs 14, 15, 16, 17, 18, 19) and electron conductivity models of aluminium have been examined through exploding wire experiments to determine the expansion rate through X-ray backlighter radiographs^20^. As the resistivity of a laboratory plasma and the viscosity of the plasma of a brown dwarf are both dependent upon electron collisions^21^ and the Coulomb logarithm, ln *Λ*, examining the resistivity models in laser-produced plasmas allows us to provide tests of the viscosity of matter found in brown dwarfs, which can be considered to be in local thermodynamic equilibrium. The laboratory measurements of return current distributions are characterized by a Maxwellian distribution of isotropic equilibrium (*f*_M_), which gives these measurements relevance to local thermodynamic equilibrium conditions, and a beam component, which is a small perturbation from the isotropic equilibrium distribution (*f*_b_), given by *f*=*f*_M_+*f*_b_cos*θ*.

In the case of brown dwarfs, the diffusion coefficient is inversely proportional to the viscosity, and as such any changes in the understanding of the viscosity model will therefore influence the temperatures of the brown dwarf. The temperature has an effect on the timescale and mixing in the radiative part of the atmosphere^22^,^23^,^24^,^25^, affecting the brown dwarf evolutionary models. As both systems are dependent on the Coulomb logarithm, a laboratory measurement of the resistivity would therefore constrain the viscosity in a brown dwarf.

In ultra-intense laser-plasma interactions, beams of relativistic ‘hot' electrons are driven from the interaction region^6^ into materials of much higher density. Anisotropies in the speed distributions of both hot and return currents generate polarized X-ray line emission. As such the experimental measurements of the degree of polarization give us a unique ability to model both the non-equilibrium anisotropy in the return current electron distribution function^12^,^13^,^26^ and in turn allows us to evaluate the resistivity generated in the warm dense matter regime applicable to the study of the viscosity of brown dwarfs. It is the non-equilibrium, or anisotropic, parts of the return current distribution that yield information about material resistivity. The return current density *J*_rc_ is related to the resistivity, *η*, by the resistive electric field *E=−ηJ*_rc_, which converts the energy of the hot electrons into Ohmic heating^16^,^27^. We can infer the resistivity from electron beam anisotropy, as the fast electrons are generated with an anisotropic distribution function, and this is enhanced by their propagation into the target^16^.

Here we demonstrate experimental measurements of the polarization of X-rays produced in intense laser-plasma interactions as a method of determining resistivity models in warm dense matter. Through doping a low-atomic number (plastic) foil target with sulphur and observing the polarization of emitted X-rays, we observe anisotropic current distributions that drive the heat transport. Our experimental measurements show a high, positive, X-ray polarization arising from anisotropic electron transport. By matching the measured polarization to detailed modelling we show that the commonly used resistivity models do not adequately describe these amorphous materials.

## Results

### Experimental set-up

**Figure 1:**
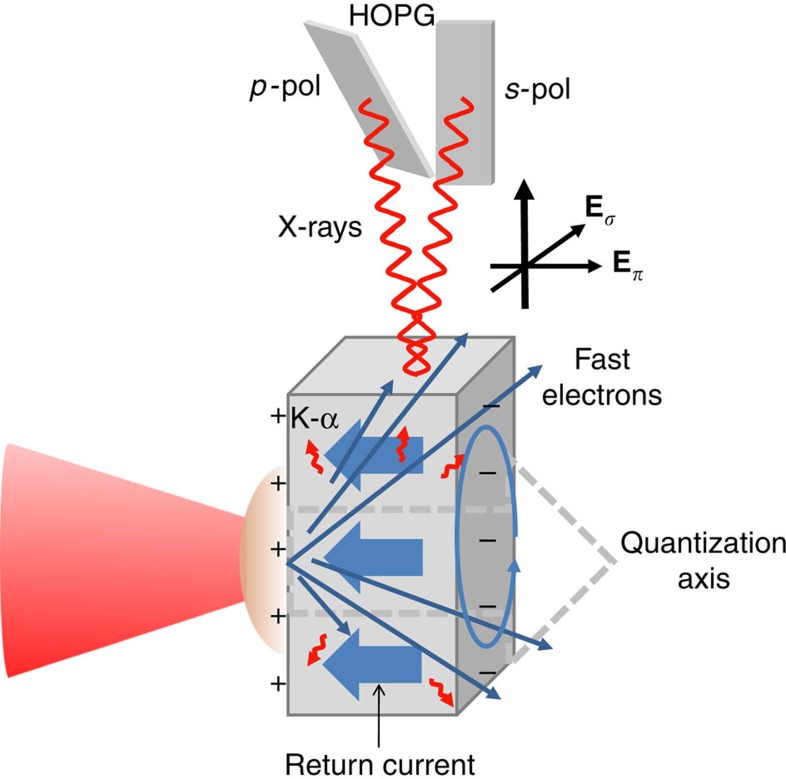
The generation of polarized X-rays in a laser-plasma interaction. The target geometry of a high-intensity pulse, incident on a foil target produces polarized X-rays. These polarized X-rays are recorded by the orthogonal pair of HOPG crystals positioned above the target (not to scale) to measure each polarization independently in a single shot.

Our experiment was performed using the Vulcan Petawatt laser at the Central Laser Facility; with on target intensities of ∼5 × 10^20^ W cm^−2^ with sulphur-doped plastic targets (polysulphone, C_27_H_26_O_6_S; for the full description of the experimental set-up, including laser parameters and diagnostics, see Fig. 1 and Methods section). The measurements were performed with an orthogonal pair of highly oriented pyrolytic graphite (HOPG) crystals at a Bragg angle, _B_=45° (where the intensity of the *p*-polarized reflection is given by *I*_p_≈*R*(*θ*)cos^2^(2_B_)), which allows us to observe time-integrated measurements of both polarizations of emitted X-rays in a single shot. The degree of polarization of X-rays emitted is calculated by 
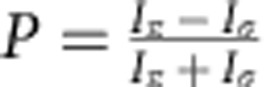
 (ref. 11), and the sign of the polarization indicates whether it arises due to beam-directional electrons with energies close to or far exceeding the excitation potential. We define a quantization axis as the direction of the free electron current. When a free electron with energy close to the excitation potential excites a ground state atomic electron, the atomic electron oscillates mostly parallel to the quantization axis. As a result, the atomic electron selects certain excited states emitting *π*-polarized dipole radiation (that is, *P*>0) as it de-excites. In contrast, an energetic free electron will exert a pulse of electric field that is perpendicular to the quantization axis causing the atomic electron to oscillate and emit mostly *σ*-polarized radiation (that is, *P*<0).

### Experimental polarization measurements

**Figure 2:**
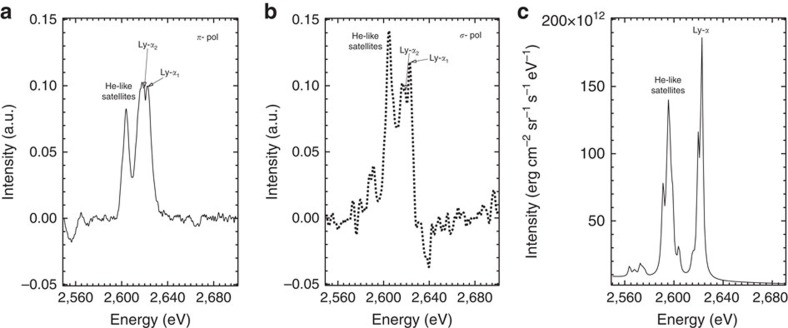
X-ray spectrum from a 25-μm-thickness sulphur-doped plastic (polysulphone) target. The two plots show the (**a**) *π*-polarized X-ray (**−**) and (**b**) *σ*-polarized X-ray (......) spectrum from the same shot. The degree of polarization observed in the He-*α* lines differ from those of the Ly-*α*, as these lines are a result of interplay between direct collisional excitation within the He-like ion as well as electron capture from the ground state^29^ and provide a future avenue for study. The third plot (**c**) is the simulated emission spectra of polysulphone modelled using the collisional-radiative spectral analysis code PrismSPECT^37^ with a temperature of *T*_b_=150 eV and *α*=0.01.

The degree of polarization in the Ly-*α* transitions of sulphur, obtained from the data shown in Fig. 2, was measured to be *P*=+0.16±0.04. This value for the polarization indicates a beam of free electrons in the return current close to the excitation potential (≥2.8 keV). This has led us to use the non-equilibrium, anisotropic, velocity distribution of the return current to explore the resistivity in warm dense plasmas. The experimental measurement of the polarization is time-integrated, and whilst laboratory plasmas do change on very fast timescales, it does not prevent a comparison with theoretical models. Uncertainties in the measurement are discussed in the Methods section.

### Simulations of the electron transport and polarization

These measurements are modelled using the ZEPHYROS^28^ and POLAR^29^ codes to calculate plasma hybrid electron kinetics and atomic magnetic sub-level population kinetics respectively. POLAR requires three electron populations and temperatures^30^ to calculate the polarization, and so by varying the input to ZEPHYROS to give us values for background temperature *T*_b_, return current temperature *T*_rc_ and hot electron fraction *α*, we can iterate between the two codes to find the conditions that most closely match the experimental observations. Using this process we find that there is a balance to be achieved between *T*_b_, *T*_rc_ and *α* that maximizes the degree of polarization. If *T*_b_ is too high, the depolarizing Maxwellian electrons will equilibrate *P* towards zero and equally, if *T*_rc_ is too low, the beam is ineffective in driving anisotropy.

**Figure 3:**
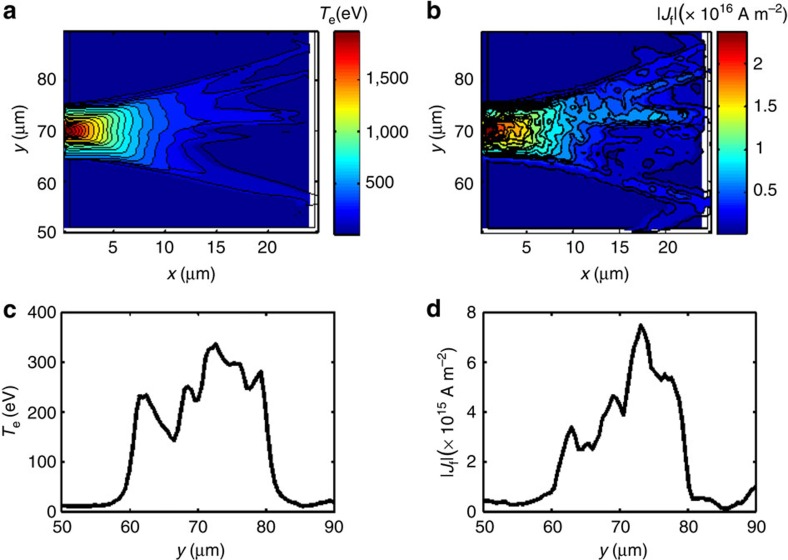
ZEPHYROS simulations of the sulphur-doped plastic target conditions. The simulations use a combination of plasma hybrid electron kinetics and atomic magnetic sub-level population kinetics to obtain the plasma parameters *T*_b_=200 eV, *T*_rc_=600 eV and *α*=0.0032. The simulation results show the simulated electron temperature (**a**) and current density (**b**) and are taken in the mid-plane of the interaction, with *x* and *y* the horizontal and vertical axes through the target respectively, and lineouts of each distribution are shown below the simulation taken through middle of the target at *x*=12.5 μm (**c** and **d** respectively). The parameters selected for the hot electron beam match the experiment and initially use the resistivity model of Davies^36^.

On taking some typical values from ZEPHYROS: *T*_b_=200 eV, *T*_rc_=600 eV and hot temperature *T*_h_=7 MeV (from Wilks' scaling^31^) with *α*=0.0032, we obtain a polarization value from the POLAR model of *P*=+0.14. The bulk temperature *T*_b_ is in the typical temperature region in the core of brown dwarfs. The ZEPHYROS simulations using the above electron distribution parameters are shown in Fig. 3. In the region shown, heating is dominated by resistive heating of the target by the anisotropic component of the return current.

**Figure 4:**
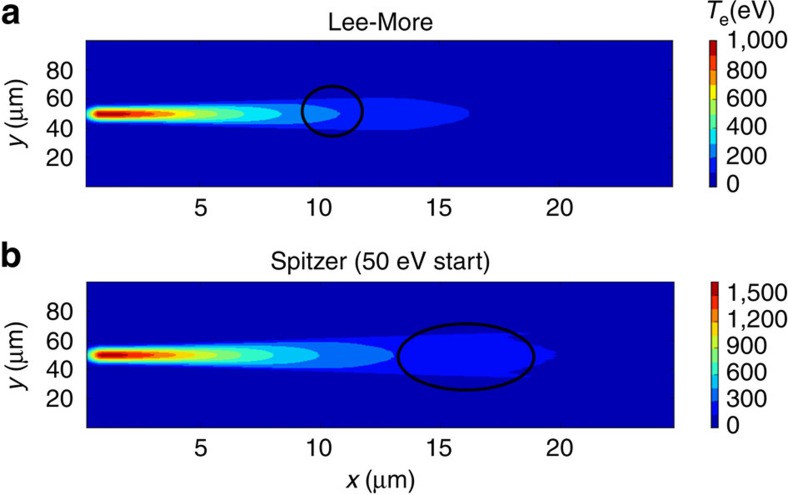
Simulations comparing the electron temperature profiles. The simulations show the plasma conditions for plasma parameters *T*_b_=200 eV, *T*_rc_=600 eV and *α*=0.0032. The simulation results are again taken in the mid-plane of the interaction, with *x* and *y* the horizontal and vertical axes through the target respectively. (**a**) shows the expected temperature profile with the commonly used Lee-More model starting with a minimum mean free path of 2 × *r*_s_. This resistivity condition produces a small volumetric region heated to 200 eV and leads to a calculated polarization of *P*=+0.275±0.1 (s.d.). Whilst it is possible that the observed polarizations are produced in this small region, the simulation from the Spitzer model with a 50 eV starting temperature in the case shown in **b** has a much larger region heated to 200 eV, which dominates over any other single temperature region, leading to a calculated polarization of *P*=+0.163±0.03 (s.d.). In both figures the region heated to 200 eV is highlighted, demonstrating that the region heated to the necessary temperature to generate the observed polarization is larger in the case of the higher resistivity Spitzer model.

To compare this model combination to the experimental results we carried out temporal and spatial averaging of the post-processed ZEPHYROS output. Two resistivity models were compared: the Lee-More^5^ model, and the Spitzer^4^ model (with an initial temperature of 50 eV). These two resistivity models primarily differ at low temperatures, where the Spitzer resistivity curve is significantly higher. The simulation results for the different resistivity models are shown in Fig. 4. The figures show the plasma material with the region with the strongest Ly-*α* emission at 2.6 keV highlighted, which arises from a region in the bulk plasma heated to approximately *T*_b_=200 eV. These plots of background electron temperature show that the target is heated to higher average temperature in the case where the Spitzer resistivity curve was employed compared with that where the Lee-More resistivity curve was used. When these results are processed with POLAR we obtained an average polarization of *P*=+0.275±0.1 for the Lee-More case and *P*=+0.163±0.03 for the Spitzer case (*T* init=50 eV). This indicates that a model with somewhat higher resistivity at low temperatures than predicted by the Lee-More model is necessary for there to be good quantitative agreement with the experimental results. The simulated polarization is sensitive to *T*_h_ for both the Lee-More and the Spitzer (*T* init=50 eV) models. For our simulations we use the Wilks scaling which gives *T*_h_=7 MeV, rather than the scaling laws of Beg^32^, Haines^33^ or Sherlock^34^ which predict lower temperatures. The calculations show Lee-More over predicts the polarization, whilst Spitzer is more consistent with our experimental result. How the polarization calculation varies with *T*_h_ for these two models are shown in Supplementary Table 1 and discussed in Supplementary Note 1. These results clearly show that conclusions of this work are not dependent on the *T*_h_ model or scaling law. Indeed a factor of >2 reduction in *T*_h_ is needed for the Lee-More model to match observation. This reduction cannot be justified using accepted scaling laws.

## Discussion

Resistivity models with a low-temperature (<100 eV) resistivity equal to or lower than the standard Lee-More model with a minimum electron scattering mean free path of 2 × *r*_s_ do not agree well with our experimental results. To be clear: our analysis is not suggesting that the Spitzer model is a ‘good' model of these plasmas. Rather it suggests that accurate computation of the resistivity at low-temperature (<100 eV) requires careful examination.

Measuring the polarization state of X-rays to give *in-situ* information about return current electron transport in warm dense matter shows a large degree of anisotropy in the return current distributions. The use of our combination of experiment and computational models allows us to observe a beam-like return current, and use this to interrogate resistivity models of warm dense matter, in this case plastic. We find a model which is highly resistive at low temperatures is necessary to obtain the level of polarization that we observe. This indicates that the resistivity in warm dense plasmas is higher than one would determine from commonly used models, and needs much more careful consideration. Since our resistivity models have to be significantly altered to understand the energy transport through plasmas, it is likely that the viscosity of the brown dwarf would also need to be modified, potentially leading to a change in temperature which would alter the evolutionary models and luminosity of these objects. With further study, through examining the resistivity of plasmas in the laboratory, it is possible to quantify the coulomb logarithm for brown dwarfs. The modelling of warm dense matter resistivity, and viscosity in the case of brown dwarfs, is so complex, it is clearly necessary to consider in more detail the resistivity curves used in such modelling.

## Methods

### Laser-target interaction

The experiment was performed with the Vulcan Petawatt system at the Central Laser Facility, Rutherford Appleton Laboratory. The laser pulses contained ∼250 J in 600 fs on target, at 1,053 nm. The beam is focussed with an *f*/3 parabola and focuses to a best spot of ≈6 × 6 μm^2^ with ∼30% of the total energy in the full width at half maximum, with peak intensities of 5 × 10^20^ W cm^−2^. The nanosecond amplified spontaneous emission contrast ratio was measured on each shot with a fast photodiode at 10^−6^.

### Targets

The targets were 25-μm-thick polysulphone foils (C_27_H_26_O_6_S) cut to 100 × 100 μm^2^ squares mounted on copper stalks.

### X-ray polarization spectrometer

Two flat, mosaic, 25 × 50 × 2 mm^3^, grade ZYA (mosaic spread 0.4°±0.1°) HOPG (002) Bragg reflecting crystals were placed above the target aligned either parallel or perpendicular to the target surface, perpendicular to the general direction of hot electrons (the quantization axis). Diffracted X-rays (with _B_∼45°) were recorded on image plates. The use of two high-reflectivity HOPG crystals was essential to enable single-shot spectroscopy measurements in an environment of high-level electromagnetic and particle noise, with relatively weak X-ray signal. (The reflectivity and rocking curves of the HOPG crystals in this context are discussed further in ref. 35).

### Experimental degree of polarization

The degree of polarization is extracted by comparing Ly-*α*_1_ integrated line intensities from the orthogonal HOPGs. To unfold the relative contributions of the Ly-*α* components and assess the level of uncertainty, a multi-line fitting procedure is applied. This procedure enables a cross calibration of the HOPG spectra by normalizing the integrated intensity of the Ly-*α*_2_ and then extracting the relative integrated intensities of the Ly-*α*_1_ emission. Sources of error and uncertainty in instrumentation and analysis are scrutinized. The experimental environment is noisy and given the crystals are positioned next to each other above the target and orientated to diffract in different directions the image plates are exposed to different levels of background. This is accounted for in the data analysis as are the uncertainty that arises from the differences in positioning.

### Numerical modelling

The plasma electron kinetics simulations have been carried out using the three-dimensional particle hybrid code ZEPHYROS^28^. The Lyman-*α* sub-level population calculations were performed with the POLAR^29^ model, which is a sub-level population, zero-dimensional atomic kinetics code. Hybrid simulations treat the hot electrons kinetically, but the background plasma is treated as a fluid. The parameters selected for the hot electron beam match the experiment and use the resistivity model of Davies' heuristic curve for CH (ref. 36), and modified to give the Spitzer resistivity for sulphur or nickel in the high-temperature limit. From this we extract spatial profiles of the hot electron current density and the ‘average' background temperature and density (*T*_b_ and *n*_b_), which are used as input to POLAR to calculate the polarization. POLAR includes background electrons as an isotropic Maxwellian population and a return current as a beamed electron population with a longitudinal temperature (*T*_rc_) and density *αn*_b_, where *α* is the return current fraction.

Errors in the calculated polarizations were calculated by performing a study varying the simulation inputs; intensity, pulse duration and absorption. These parameters were varied between upper and lower limits of possible experimental values, to find the likelihood of errors between the experimental data and our simulated polarizations.

## Additional information

**How to cite this article:** Booth, N. *et al*. Laboratory measurements of resistivity in warm dense plasmas relevant to the microphysics of brown dwarfs. *Nat. Commun.* 6:8742 doi: 10.1038/ncomms9742 (2015).

## Supplementary Material

Supplementary Information Supplementary Table 1, Supplementary Note 1 and Supplementary References

## References

[b1] Chabrier G. & Baraffe I. Theory of low-mass stars and substellar objects. Ann. Rev. Astron. Astrophys. 38, 337–377 (2000).

[b2] Ichimaru S. & Kitamura H. Pyconuclear reactions in dense astrophysical and fusion plasmas. Phys. Plasmas 6, 2649–2671 (1999).

[b3] Baraffe I., Chabrier G. & Barman T. The physical properties of extra-solar planets. Rep. Prog. Phys. 73, 016901 (2010).

[b4] Spitzer L. & Harm R. Transport phenomena in a completely ionized gas. Phys. Rev. 89, 977–981 (1953).

[b5] Lee Y. T. & More R. M. An electron conductivity model for dense plasmas. Phys. Fluids 27, 1273–1286 (1984).

[b6] Bell A. R., Davies J. R., Guerin S. & Ruhl H. Fast electron transport in high-intensity, short-pulse laser-solid experiments. Plasma Phys. Control. Fusion 39, 653–659 (1997).

[b7] Evans R. G. Modelling electron transport for fast ignition. Plasma Phys. Control. Fusion 49, B87–B93 (2007).

[b8] Burrows A. & Liebert J. The science of brown dwarfs. Rev. Mod. Phys. 65, 301–336 (1993).

[b9] Saumon D., Chabrier G. & Van Horn H. M. An equation of state for low-mass stars and giant planets. Astrophys. J. Suppl. 99, 713–741 (1995).

[b10] Remington B. A., Arnett D., Drake R. P. & Takabe H. Modelling astrophysical phenomena in the laboratory with intense lasers. Science 284, 1488–1493 (1999).

[b11] Kieffer J. C. . Electron distribution anisotropy in laser-produced plasmas from x-ray line polarization measurements. Phys. Rev. Lett. 68, 480–483 (1992). 10045907 10.1103/PhysRevLett.68.480

[b12] Inubushi Y. . X-ray line polarization spectroscopy to study hot electron transport in ultra-short laser produced plasma. J. Quant. Spectrosc. Radiat. Transf. 99, 305–313 (2006).

[b13] Kawamura T. . Polarization of He-α radiation due to anisotropy in fast electron transport in ultra-intense laser-produced plasmas. Phys. Rev. Lett. 99, 115003 (2007). 17930447 10.1103/PhysRevLett.99.115003

[b14] Milchberg H. M., Freeman R. R. & Davey S. C. Resistivity of a simple metal from room temperature to 10^6^ K. Phys. Rev. Lett. 61, 2364–2367 (1988). 10039093 10.1103/PhysRevLett.61.2364

[b15] Davies J. R., Bell A. R. & Tatarakis M. Magnetic focussing and trapping of high-intensity laser-generated fast electrons at the rear of solid targets. Phys. Rev. E 59, 6032–6036 (1999). 10.1103/physreve.59.6032 11969587

[b16] Bell A. R. & Kingham R. J. Resistive collimation of electron beams in laser produced plasmas. Phys. Rev. Lett. 91, 035003 (2003). 12906424 10.1103/PhysRevLett.91.035003

[b17] MacLellan D. A. . Annular fast electron transport in silicon arising from low-temperature resistivity. Phys. Rev. Lett. 111, 095001 (2013). 24033041 10.1103/PhysRevLett.111.095001

[b18] DeSilva A. W. & Katsouros J. D. Electrical conductivity of dense copper and aluminum plasmas. Phys. Rev. E 57, 5945–5951 (1998).

[b19] Brown C. R. D. . Measurements of electron transport in foils irradiated with a picosecond timescale laser pulse. Phys. Rev. Lett. 106, 185003 (2011). 21635097 10.1103/PhysRevLett.106.185003

[b20] Sinars D. B. . Exploding aluminium wire expansion rate with 1-4.5kA per wire. Phys. Plasmas 7, 1555–1563 (2000).

[b21] Mohanty S. . Activity in very cool stars: Magnetic dissipation in late M and L dwarf atmospheres. Astrophys. J. 571, 469–486 (2002).

[b22] Saumon D. . Physical parameters of very cool T dwarfs. Astrophys. J. 656, 1136–1149 (2007).

[b23] Leggett S. K. . The physical properties of four ∼600K T dwarfs. Astrophys. J. 695, 1517–1526 (2009).

[b24] Stephens D. C. . The 0.8 – 14.5μm spectra of mid-L to mid-T dwarfs: diagnostics of effective temperature, grain sedimentation, gas transport and surface gravity. Astrophys. J. 702, 154–170 (2009).

[b25] Leggett S. K. . Properties of the T8.5 dwarf wolf 940B. Astrophys. J. 720, 252–258 (2010).

[b26] Inubushi Y. . Analysis of x-ray polarization to determine three-dimensionally anisotropic velocity distributions of hot electrons in plasma produced by ultrahigh intensity lasers. Phys. Rev. E 75, 026401 (2007). 10.1103/PhysRevE.75.026401 17358426

[b27] Huang X. & Cumming A. Ohmic dissipation in the interiors of hot Jupiters. Astrophys. J. 757, 47 (2012).

[b28] Kar S. . Guiding of relativisitic electron beams in solid targets by resistively controlled magnetic fields. Phys. Rev. Lett. 102, 055001 (2009). 19257515 10.1103/PhysRevLett.102.055001

[b29] Hakel P. & Mancini R. C. X-ray line polarization of He-like Si satellite spectra in plasmas driven by high-intensity ultrashort pulsed lasers. Phys. Rev. E 69, 056405 (2004). 10.1103/PhysRevE.69.056405 15244949

[b30] Hakel P. Polarization properties of the Ly-α line from sulphur plasmas driven by high-intensity, ultrashort-duration laser pulses. Can. J. Phys. 89, 509–511 (2011).

[b31] Wilks S. C. & Kruer W. L. Absorption of ultrashort, ultra-intense laser light by solids and overdense plasmas. IEEE J. Quantum Electron. 33, 1954–1968 (1997).

[b32] Beg F. N. . A study of picosecond laser–solid interactions up to 1 × 10^19^ W cm^−2^. Phys. Plamas 4, 447–457 (1997).

[b33] Haines M. G. . Hot-electron temperature and laser-light absorption in fast ignition. Phys. Rev. Lett. 102, 045008 (2009). 19257435 10.1103/PhysRevLett.102.045008

[b34] Sherlock M. Universal scaling of the electron distribution function in one-dimensional simulations of relativistic laser-plasma interactions. Phys. Plasmas 16, 103101 (2009).

[b35] Woolsey N. C. . Precision x-ray spectroscopy of intense laser-plasma interactions. High Energy Density Phys. 7, 105–109 (2011).

[b36] Davies J. R. How wrong is collisional Monte Carlo modelling of fast electron transport in high-intensity, laser-solid interactions? Phys. Rev. E 65, 026407 (2002). 10.1103/PhysRevE.65.026407 11863668

[b37] McFarlane J. J. . Simulation of the ionization dynamics of aluminum irradiated by intense short-pulse lasers. *Proc. Inertial Fusion and Sciences Applications 2003*. Amer. Nucl. Soc. 457–469 (2004).

