# Supplementary material for: Laboratory measurements of resistivity in warm dense plasmas relevant to the microphysics of brown dwarfs
Source: Nat Commun. 2015 Nov 6;6:8742. doi: 10.1038/ncomms9742 (PMC4667641; doi:10.1038/ncomms9742)
Supplement: Supplementary Information — Supplementary Table 1, Supplementary Note 1 and Supplementary References [file ncomms9742-s1.pdf]

Supplementary Table 1

Table 1: Values of the average polarisation $<P>$ for cases of T_h as a fraction/multiple of T_{pond} for the Lee-More models and Spitzer (starting with an initial temperature at 50eV).

T_h	$<P>$ from Lee-More model simulations	$<P>$ from Spitzer (T init at 50eV) model simulations
0.4 T_h	0.172	0.086
0.6 T_h	0.217	0.118
0.8 T_h	0.248	0.141
1.2 T_h	0.294	0.182

Supplementary Notes 1

For the simulations presented in our paper in figures 3 and 4, we began by using the standard Spitzer model to give us the initial parameters for the bulk and return current temperatures, and the absorption fraction, then used this as a starting point to make modifications to the resistivity model. A number of modified resistivity models were used in the initial simulations, and the standard Lee-More and the Spitzer with the modified curve at lower temperatures were selected to best demonstrate our point – not that Spitzer is correct, rather careful examination of the resistivity models is needed at lower temperatures.

Previous work has discussed the issues that can be seen with the Lee-More model as a universal model for resistivity of highly crystalline carbon and silicon targets[1,2]. What we present here is evidence that there is the potential for issues with the low temperature resistivity even in the case where the material is amorphous or disordered. In table 1 we report the values obtained from examining the sensitivity to the choice of T_h in the numerical simulations.

Calculations in table 1 show that the Lee-More model would predict high polarisation values. There is no experimental evidence for this. In contrast the Spitzer model with an initial temperature of 50 eV yields polarisation results which are more in line with measurement although the Spitzer model result does under or over predict the polarisation for modest changes to the fast electron temperature. The experiment result is P=+0.16±0.04

The key point is that with some modest variation in the exact value of the fast electron temperature the Spitzer model results yield results that compare more favourably with the experiment than the Lee-More model. That the Lee-More model provides a reasonable value for unrealistically low fast electron temperature is unlikely. We conclude the Lee-More model does not represent the plasma resistivity. Simulation studies indicate that where there is a 'sub-ponderomotive' part of the energy spectrum there is almost always a hotter component that carries most of the absorbed laser energy[3,4,5]. We therefore conclude that such a low temperature beam is not responsible for driving the polarised x-ray emission.

Supplementary References

1. McKenna, P. et al, Effect of Lattice structure on energetic electron transport in solids irradiated by ultraintense laser pulses, Phys. Rev. Letts, **106**, 185004 (2011)

2. MacLellan, D.A. et al, Annular fast electron transport in silicon arising from low-temperature resistivity, Phys. Rev. Letts, **111**, 095001 (2013)

3. Haines, M.G. et al, Hot-electron temperature and laser-light absorption in fast ignition, Phys. Rev. Letts, **102** 045008 (2009)

4. Sherlock, M. Universal scaling of the electron distribution function in one-dimensional simulations of relativistic laser-plasma interactions, Phys. Plasmas, **16**, 103101 (2009)

5. Robinson, A.P.L and Schmitz, H., Elliptical magnetic mirror generated via resistivity gradients for fast ignition inertial confinement fusion, Phys. Plasmas, **20**, 062704 (2013)
